# μ-Oxido-bis­[(2-chloro­nicotinato-κ*O*)triphenyl­anti­mony(V)]

**DOI:** 10.1107/S1600536808042335

**Published:** 2008-12-20

**Authors:** Li Quan, Handong Yin, Daqi Wang

**Affiliations:** a College of Chemistry and Chemical Engineering, Liaocheng University, Shandong 252059, People’s Republic of China

## Abstract

A new dinuclear triphenyl­anti­mony(V) derivative with an oxide bridge, [Sb_2_(C_6_H_5_)_6_(C_6_H_3_ClNO_2_)_2_O], has been synthesized. Each Sb atom is five-coordianted by three C atoms and two O atoms in a distorted trigonal-bipyramidal geometry. Metal centers are bridged by a μ_2_-oxide functionality, and phenyl substituents on Sb atoms are in an staggered arrangement. The Sb—O—Sb bridge displays a bent geometry with an angle of 165.1 (4)°. Mol­ecules inter­act in the crystal through weak C—H⋯O and C—H⋯N inter­molecular hydrogen bonds.

## Related literature

For the synthesis and structures of related triphenyl­anti­mony compounds, see: Ferguson & Ridley (1973 ▶); Preut *et al.* (1985 ▶, 1986 ▶).
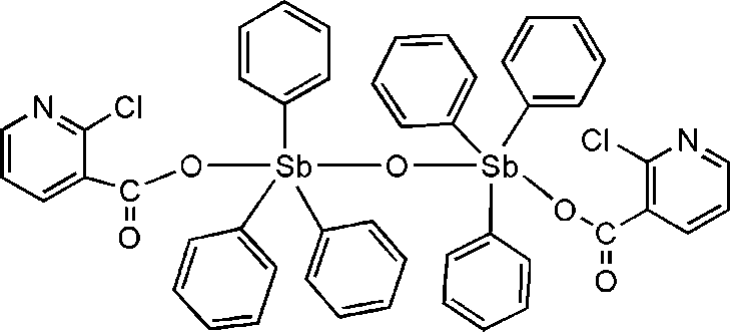


## Experimental

### 

#### Crystal data

[Sb_2_(C_6_H_5_)_6_(C_6_H_3_ClNO_2_)_2_O]
*M* *_r_* = 1035.19
Monoclinic, 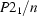

*a* = 20.477 (2) Å
*b* = 9.6220 (11) Å
*c* = 22.513 (3) Å
β = 94.978 (2)°
*V* = 4419.0 (9) Å^3^
*Z* = 4
Mo *K*α radiation
μ = 1.39 mm^−1^
*T* = 298 (2) K
0.45 × 0.26 × 0.19 mm

#### Data collection

Bruker SMART diffractometer
Absorption correction: multi-scan (*SADABS*; Sheldrick, 1996 ▶) *T*_min_ = 0.567, *T*_max_ = 0.770
20773 measured reflections
7764 independent reflections
4921 reflections with *I* > 2σ(*I*)
*R*_int_ = 0.083

#### Refinement

*R*[*F* ^2^ > 2σ(*F* ^2^)] = 0.062
*wR*(*F* ^2^) = 0.135
*S* = 1.05
7764 reflections
532 parameters
H-atom parameters constrained
Δρ_max_ = 2.07 e Å^−3^
Δρ_min_ = −1.02 e Å^−3^

### 

Data collection: *SMART* (Siemens, 1996 ▶); cell refinement: *SAINT* (Siemens, 1996 ▶); data reduction: *SAINT*; program(s) used to solve structure: *SHELXS97* (Sheldrick, 2008 ▶); program(s) used to refine structure: *SHELXL97* (Sheldrick, 2008 ▶); molecular graphics: *DIAMOND* (Brandenburg, 1998 ▶); software used to prepare material for publication: *SHELXL97*.

## Supplementary Material

Crystal structure: contains datablocks I, global. DOI: 10.1107/S1600536808042335/bh2210sup1.cif

Structure factors: contains datablocks I. DOI: 10.1107/S1600536808042335/bh2210Isup2.hkl

Additional supplementary materials:  crystallographic information; 3D view; checkCIF report

## Figures and Tables

**Table:** 

Sb1—O1	1.955 (5)
Sb1—O2	2.208 (5)
Sb2—O1	1.955 (5)
Sb2—O4	2.229 (5)

**Table:** 

O1—Sb1—O2	179.2 (2)
O1—Sb2—O4	174.6 (2)
Sb1—O1—Sb2	165.1 (4)

**Table 2:** Hydrogen-bond geometry (Å, °)

*D*—H⋯*A*	*D*—H	H⋯*A*	*D*⋯*A*	*D*—H⋯*A*
C17—H17⋯N2^i^	0.93	2.66	3.556 (14)	163
C39—H39⋯O5^ii^	0.93	2.63	3.544 (12)	167
C23—H23⋯O3^iii^	0.93	2.37	3.166 (12)	144
C23—H23⋯O3^iii^	0.93	2.37	3.166 (12)	144

Symmetry codes: (i) 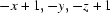; (ii) 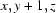; (iii) 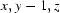.

**Fig. 1.**
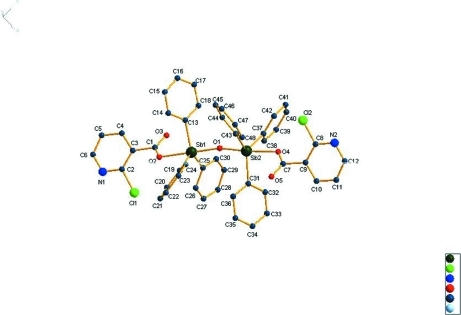
The molecular structure of the title compound, showing displacement ellipsoids at the 30% probability level.

**Fig. 2.**
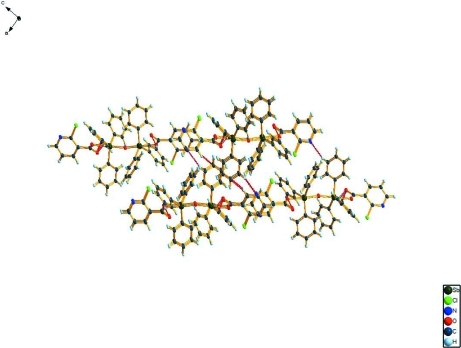
A 3D network of hydrogen bonds in the crystal structure of the title compound. Symmetry code: (i) x, y+1, z; (ii) -x+1, -y, -z+1; (iii) x, y-1, z; (iv) x, y-1, z.

## supplementary crystallographic information

### Comment

In the title complex, two sets of Sb—O bonds are observed, of which the bridging bond Sb1—O1, 1.955 (5) Å, is the shortest and correspond to the boundary for known range of Sb—O bond lengths in (Ph_3_SbX)_2_O species. The shortest coordinating Sb—O bond, Sb1—O2 = 2.208 (5) Å, is associated with the longest C—O bond, C1—O2 = 1.314 (8). In contrast, the long Sb2—O4 bond length, 2.229 (5) Å, is associated to the short C—O bond, C7—O4 = 1.251 (9) Å (Ferguson & Ridley, 1973; Preut *et al.*, 1985, 1986). In the crystal structure, the molecules are connected through weak C—H···O and C—H···N intermolecular hydrogen bonds (Fig. 2.)

### Experimental

2-Chloropyridyl-3-carboxylic acid (0.315 g, 2 mmol) was dissolved in dry toluene (15 ml) together with triethylamine (0.202 g, 2 mmol), and the mixture was refluxed for 30 min. Then µ-oxo-bis(triphenylantimony(V)-chloride) (0.423 g, 1 mmol) dissolved in toluene (15 ml) was added. The reaction was allowed to complete for 12 h at room temperature. After filtration, the solvent was gradually removed by evaporation under vacuum, until a white solid was obtained. The solid was recrystallized from petroleum ether/dichloromethane (1:1) to give colourless crystals.

### Refinement

All H atoms were positioned geometrically and refined as riding atoms, with C—H = 0.93 Å and *U*_iso_(H) = 1.2*U*_eq_(carrier C).

### Crystal data

**Table:**

[Sb_2_(C_6_H_5_)_6_(C_6_H_3_ClNO_2_)_2_O]	*F*(000) = 2056
*M**_r_* = 1035.19	*D*_x_ = 1.556 Mg m^−^^3^
Monoclinic, *P*2_1_/*n*	Mo *K*α radiation, λ = 0.71073 Å
Hall symbol: -P 2yn	Cell parameters from 7208 reflections
*a* = 20.477 (2) Å	θ = 2.3–27.7°
*b* = 9.6220 (11) Å	µ = 1.39 mm^−^^1^
*c* = 22.513 (3) Å	*T* = 298 K
β = 94.978 (2)°	Block, colourless
*V* = 4419.0 (9) Å^3^	0.45 × 0.26 × 0.19 mm
*Z* = 4	

### Data collection

**Table:**

Bruker SMART diffractometer	7764 independent reflections
Radiation source: fine-focus sealed tube	4921 reflections with *I* > 2σ(*I*)
graphite	*R*_int_ = 0.083
φ and ω scans	θ_max_ = 25.0°, θ_min_ = 1.4°
Absorption correction: multi-scan (*SADABS*; Sheldrick, 1996)	*h* = −20→24
*T*_min_ = 0.567, *T*_max_ = 0.770	*k* = −10→11
20773 measured reflections	*l* = −17→26

### Refinement

**Table:**

Refinement on *F*^2^	Primary atom site location: structure-invariant direct methods
Least-squares matrix: full	Secondary atom site location: difference Fourier map
*R*[*F*^2^ > 2σ(*F*^2^)] = 0.062	Hydrogen site location: inferred from neighbouring sites
*wR*(*F*^2^) = 0.135	H-atom parameters constrained
*S* = 1.05	*w* = 1/[σ^2^(*F*_o_^2^) + (0.045*P*)^2^ + 6.3977*P*] where *P* = (*F*_o_^2^ + 2*F*_c_^2^)/3
7764 reflections	(Δ/σ)_max_ = 0.001
532 parameters	Δρ_max_ = 2.07 e Å^−^^3^
0 restraints	Δρ_min_ = −1.02 e Å^−^^3^

### Fractional atomic coordinates and isotropic or equivalent isotropic displacement parameters (Å^2^)

**Table:**

	*x*	*y*	*z*	*U*_iso_*/*U*_eq_	
Sb1	0.44005 (2)	0.10749 (5)	0.19403 (2)	0.03649 (16)	
Sb2	0.55296 (2)	−0.06381 (5)	0.32056 (2)	0.03710 (17)	
Cl1	0.40604 (13)	0.2694 (4)	−0.01255 (13)	0.1010 (10)	
Cl2	0.60168 (11)	−0.2946 (3)	0.51028 (12)	0.0704 (7)	
N1	0.2954 (4)	0.3827 (9)	−0.0438 (4)	0.082 (3)	
N2	0.7167 (4)	−0.3926 (8)	0.5378 (4)	0.073 (2)	
O1	0.4918 (2)	0.0377 (6)	0.2648 (2)	0.0554 (15)	
O2	0.3813 (2)	0.1832 (5)	0.1136 (2)	0.0391 (13)	
O3	0.3645 (3)	0.3807 (6)	0.1607 (3)	0.0614 (16)	
O4	0.6211 (2)	−0.1640 (5)	0.3904 (2)	0.0383 (13)	
O5	0.6467 (3)	−0.3492 (6)	0.3376 (3)	0.0603 (16)	
C1	0.3584 (4)	0.3045 (9)	0.1167 (4)	0.042 (2)	
C2	0.3316 (4)	0.3395 (10)	0.0038 (4)	0.064 (3)	
C3	0.3172 (4)	0.3516 (9)	0.0621 (4)	0.047 (2)	
C4	0.2598 (4)	0.4261 (9)	0.0693 (5)	0.065 (3)	
H4	0.2479	0.4447	0.1074	0.079*	
C5	0.2209 (5)	0.4719 (11)	0.0205 (6)	0.088 (4)	
H5	0.1823	0.5203	0.0250	0.105*	
C6	0.2401 (6)	0.4449 (12)	−0.0338 (6)	0.095 (4)	
H6	0.2124	0.4720	−0.0667	0.114*	
C7	0.6508 (4)	−0.2755 (9)	0.3831 (3)	0.042 (2)	
C8	0.6795 (4)	−0.3396 (8)	0.4927 (4)	0.051 (2)	
C9	0.6964 (4)	−0.3276 (9)	0.4352 (4)	0.050 (2)	
C10	0.7589 (5)	−0.3705 (11)	0.4241 (5)	0.080 (3)	
H10	0.7735	−0.3623	0.3863	0.095*	
C11	0.7982 (6)	−0.4250 (14)	0.4703 (6)	0.112 (5)	
H11	0.8400	−0.4564	0.4639	0.135*	
C12	0.7765 (6)	−0.4336 (13)	0.5256 (6)	0.098 (4)	
H12	0.8046	−0.4696	0.5565	0.117*	
C13	0.3583 (3)	0.1332 (8)	0.2427 (3)	0.0380 (18)	
C14	0.3002 (4)	0.0707 (11)	0.2244 (4)	0.073 (3)	
H14	0.2964	0.0215	0.1887	0.087*	
C15	0.2476 (5)	0.0793 (15)	0.2578 (6)	0.100 (4)	
H15	0.2092	0.0313	0.2461	0.120*	
C16	0.2516 (6)	0.1569 (13)	0.3072 (6)	0.087 (4)	
H16	0.2151	0.1667	0.3288	0.104*	
C17	0.3087 (7)	0.2217 (11)	0.3261 (5)	0.092 (4)	
H17	0.3111	0.2752	0.3606	0.111*	
C18	0.3628 (5)	0.2086 (9)	0.2947 (4)	0.068 (3)	
H18	0.4022	0.2504	0.3084	0.082*	
C19	0.4400 (3)	−0.0780 (7)	0.1436 (4)	0.040 (2)	
C20	0.4469 (4)	−0.0785 (10)	0.0843 (4)	0.064 (3)	
H20	0.4515	0.0042	0.0638	0.077*	
C21	0.4468 (5)	−0.2042 (13)	0.0552 (6)	0.093 (4)	
H21	0.4517	−0.2061	0.0145	0.112*	
C22	0.4396 (5)	−0.3254 (13)	0.0847 (8)	0.098 (5)	
H22	0.4401	−0.4089	0.0640	0.118*	
C23	0.4317 (5)	−0.3272 (11)	0.1441 (7)	0.093 (4)	
H23	0.4260	−0.4107	0.1638	0.112*	
C24	0.4322 (4)	−0.2014 (9)	0.1747 (5)	0.069 (3)	
H24	0.4273	−0.1997	0.2154	0.082*	
C25	0.5104 (5)	0.2593 (12)	0.1798 (5)	0.086 (4)	
C26	0.5370 (5)	0.2649 (12)	0.1250 (5)	0.096 (4)	
H26	0.5226	0.2033	0.0948	0.116*	
C27	0.5848 (5)	0.3614 (12)	0.1152 (5)	0.098 (4)	
H27	0.6035	0.3623	0.0791	0.117*	
C28	0.6043 (5)	0.4540 (12)	0.1576 (5)	0.093 (4)	
H28	0.6373	0.5173	0.1512	0.112*	
C29	0.5762 (5)	0.4552 (11)	0.2093 (6)	0.094 (4)	
H29	0.5886	0.5212	0.2382	0.113*	
C30	0.5281 (5)	0.3567 (10)	0.2195 (5)	0.078 (3)	
H30	0.5081	0.3598	0.2551	0.094*	
C31	0.6260 (4)	−0.0831 (8)	0.2614 (3)	0.0419 (19)	
C32	0.6865 (5)	−0.0301 (13)	0.2772 (5)	0.099 (4)	
H32	0.6946	0.0134	0.3140	0.119*	
C33	0.7358 (6)	−0.0394 (13)	0.2399 (6)	0.098 (4)	
H33	0.7758	0.0044	0.2494	0.117*	
C34	0.7250 (6)	−0.1142 (14)	0.1886 (6)	0.100 (4)	
H34	0.7594	−0.1306	0.1652	0.120*	
C35	0.6649 (6)	−0.1646 (11)	0.1715 (5)	0.086 (3)	
H35	0.6573	−0.2093	0.1349	0.103*	
C36	0.6140 (4)	−0.1501 (9)	0.2084 (4)	0.064 (3)	
H36	0.5726	−0.1856	0.1969	0.077*	
C37	0.5658 (3)	0.1034 (8)	0.3808 (4)	0.0404 (19)	
C38	0.5912 (5)	0.2259 (9)	0.3622 (5)	0.070 (3)	
H38	0.6013	0.2367	0.3230	0.084*	
C39	0.6016 (6)	0.3336 (10)	0.4028 (7)	0.096 (4)	
H39	0.6192	0.4170	0.3908	0.115*	
C40	0.5864 (5)	0.3187 (11)	0.4600 (6)	0.086 (3)	
H40	0.5939	0.3919	0.4867	0.103*	
C41	0.5608 (5)	0.1993 (10)	0.4783 (5)	0.073 (3)	
H41	0.5494	0.1901	0.5172	0.087*	
C42	0.5515 (4)	0.0896 (8)	0.4382 (4)	0.054 (2)	
H42	0.5353	0.0054	0.4510	0.065*	
C43	0.4813 (4)	−0.2111 (8)	0.3396 (3)	0.0369 (18)	
C44	0.4183 (4)	−0.1642 (10)	0.3414 (5)	0.073 (3)	
H44	0.4081	−0.0716	0.3331	0.088*	
C45	0.3699 (5)	−0.2554 (13)	0.3558 (5)	0.088 (4)	
H45	0.3271	−0.2236	0.3567	0.105*	
C46	0.3837 (5)	−0.3904 (12)	0.3687 (5)	0.080 (3)	
H46	0.3506	−0.4510	0.3778	0.096*	
C47	0.4452 (5)	−0.4351 (10)	0.3680 (5)	0.082 (3)	
H47	0.4551	−0.5272	0.3777	0.099*	
C48	0.4944 (4)	−0.3475 (9)	0.3532 (4)	0.067 (3)	
H48	0.5369	−0.3812	0.3525	0.080*	

### Atomic displacement parameters (Å^2^)

**Table:**

	*U*^11^	*U*^22^	*U*^33^	*U*^12^	*U*^13^	*U*^23^
Sb1	0.0423 (3)	0.0373 (3)	0.0291 (3)	−0.0034 (2)	−0.0012 (2)	0.0032 (2)
Sb2	0.0417 (3)	0.0410 (3)	0.0281 (3)	−0.0049 (2)	0.0002 (2)	0.0047 (2)
Cl1	0.0790 (18)	0.172 (3)	0.0542 (18)	0.0339 (19)	0.0204 (15)	0.0190 (19)
Cl2	0.0636 (14)	0.0895 (18)	0.0606 (17)	0.0100 (13)	0.0190 (13)	0.0172 (14)
N1	0.082 (6)	0.105 (7)	0.056 (6)	0.017 (5)	−0.008 (5)	0.021 (5)
N2	0.080 (6)	0.087 (6)	0.049 (5)	0.011 (5)	−0.007 (5)	0.014 (5)
O1	0.052 (3)	0.066 (4)	0.046 (4)	−0.011 (3)	−0.012 (3)	0.022 (3)
O2	0.051 (3)	0.033 (3)	0.032 (3)	0.003 (2)	−0.005 (3)	0.003 (2)
O3	0.087 (4)	0.045 (4)	0.052 (4)	0.000 (3)	0.005 (3)	0.001 (3)
O4	0.052 (3)	0.029 (3)	0.033 (3)	0.004 (2)	−0.003 (3)	0.005 (2)
O5	0.079 (4)	0.057 (4)	0.044 (4)	0.005 (3)	0.000 (3)	−0.002 (3)
C1	0.047 (5)	0.054 (6)	0.025 (5)	−0.009 (4)	0.003 (4)	0.006 (4)
C2	0.053 (5)	0.081 (7)	0.055 (7)	0.009 (5)	−0.002 (5)	0.019 (5)
C3	0.040 (5)	0.076 (6)	0.027 (5)	−0.012 (4)	0.007 (4)	0.000 (4)
C4	0.057 (6)	0.061 (6)	0.077 (7)	0.015 (5)	0.001 (5)	0.006 (5)
C5	0.074 (7)	0.075 (8)	0.110 (11)	0.032 (6)	−0.012 (7)	0.010 (8)
C6	0.097 (9)	0.108 (10)	0.073 (9)	0.022 (7)	−0.036 (8)	0.021 (8)
C7	0.052 (5)	0.053 (6)	0.020 (5)	−0.006 (4)	−0.002 (4)	0.003 (4)
C8	0.056 (5)	0.053 (5)	0.044 (6)	0.005 (4)	−0.001 (5)	0.002 (4)
C9	0.050 (5)	0.053 (5)	0.045 (6)	0.005 (4)	−0.002 (4)	0.000 (4)
C10	0.068 (6)	0.120 (9)	0.052 (7)	0.031 (6)	0.011 (5)	0.003 (6)
C11	0.085 (8)	0.163 (13)	0.085 (10)	0.069 (8)	−0.011 (8)	−0.006 (9)
C12	0.082 (8)	0.136 (11)	0.070 (9)	0.049 (7)	−0.017 (7)	0.010 (8)
C13	0.045 (4)	0.041 (5)	0.028 (5)	0.000 (4)	0.007 (4)	0.003 (4)
C14	0.053 (5)	0.121 (9)	0.045 (6)	−0.019 (6)	0.004 (5)	−0.008 (6)
C15	0.058 (7)	0.168 (13)	0.076 (9)	−0.023 (7)	0.013 (6)	0.021 (9)
C16	0.072 (8)	0.116 (10)	0.077 (9)	0.027 (7)	0.034 (7)	0.028 (8)
C17	0.136 (11)	0.072 (8)	0.076 (9)	0.008 (7)	0.051 (9)	−0.012 (7)
C18	0.088 (7)	0.064 (6)	0.058 (7)	−0.023 (5)	0.032 (6)	−0.020 (5)
C19	0.041 (4)	0.028 (5)	0.051 (6)	0.000 (3)	−0.005 (4)	−0.002 (4)
C20	0.069 (6)	0.072 (7)	0.054 (7)	0.003 (5)	0.014 (5)	−0.017 (6)
C21	0.094 (8)	0.090 (9)	0.098 (10)	0.010 (7)	0.028 (7)	−0.050 (8)
C22	0.081 (8)	0.059 (8)	0.156 (15)	−0.002 (6)	0.015 (9)	−0.050 (10)
C23	0.103 (9)	0.046 (7)	0.131 (13)	−0.014 (6)	0.019 (9)	−0.015 (8)
C24	0.068 (6)	0.055 (6)	0.083 (8)	−0.014 (5)	0.007 (6)	−0.012 (6)
C25	0.088 (7)	0.116 (9)	0.056 (7)	−0.058 (7)	0.015 (6)	−0.007 (7)
C26	0.098 (8)	0.126 (10)	0.067 (8)	−0.060 (7)	0.020 (7)	−0.010 (7)
C27	0.100 (8)	0.129 (10)	0.067 (8)	−0.058 (8)	0.021 (7)	−0.007 (8)
C28	0.094 (8)	0.122 (10)	0.065 (8)	−0.060 (7)	0.018 (7)	−0.006 (8)
C29	0.118 (9)	0.069 (7)	0.095 (10)	−0.049 (7)	0.014 (8)	−0.016 (7)
C30	0.087 (7)	0.080 (7)	0.072 (8)	−0.031 (6)	0.028 (6)	−0.017 (6)
C31	0.045 (4)	0.051 (5)	0.031 (5)	−0.011 (4)	0.009 (4)	0.007 (4)
C32	0.056 (6)	0.190 (13)	0.054 (7)	−0.041 (7)	0.022 (6)	−0.008 (8)
C33	0.096 (9)	0.131 (11)	0.068 (8)	−0.055 (8)	0.019 (7)	−0.006 (8)
C34	0.068 (8)	0.151 (12)	0.088 (10)	0.004 (8)	0.040 (7)	0.005 (9)
C35	0.115 (9)	0.096 (8)	0.052 (7)	0.003 (7)	0.038 (7)	−0.010 (6)
C36	0.069 (6)	0.073 (7)	0.053 (7)	−0.016 (5)	0.018 (5)	−0.012 (5)
C37	0.047 (4)	0.036 (5)	0.038 (5)	−0.001 (4)	0.004 (4)	−0.001 (4)
C38	0.099 (8)	0.036 (5)	0.078 (8)	−0.008 (5)	0.015 (6)	0.005 (5)
C39	0.135 (10)	0.028 (6)	0.128 (12)	−0.018 (6)	0.028 (9)	0.006 (7)
C40	0.103 (9)	0.054 (7)	0.101 (11)	−0.003 (6)	0.018 (8)	−0.025 (7)
C41	0.090 (7)	0.069 (7)	0.062 (7)	−0.011 (6)	0.022 (6)	−0.027 (6)
C42	0.069 (6)	0.040 (5)	0.056 (6)	−0.012 (4)	0.014 (5)	−0.007 (5)
C43	0.051 (5)	0.047 (5)	0.013 (4)	−0.008 (4)	0.003 (3)	0.001 (4)
C44	0.059 (6)	0.066 (6)	0.097 (9)	−0.007 (5)	0.023 (6)	0.011 (6)
C45	0.056 (6)	0.107 (10)	0.105 (10)	−0.024 (6)	0.035 (6)	−0.002 (8)
C46	0.080 (8)	0.083 (8)	0.079 (8)	−0.048 (7)	0.019 (6)	0.006 (7)
C47	0.094 (8)	0.055 (6)	0.098 (9)	−0.032 (6)	0.007 (7)	0.022 (6)
C48	0.060 (6)	0.062 (6)	0.076 (8)	−0.012 (5)	0.001 (5)	0.009 (6)

### Geometric parameters (Å, °)

**Table:**

Sb1—O1	1.955 (5)	C21—C22	1.356 (16)
Sb1—C13	2.094 (7)	C21—H21	0.9300
Sb1—C25	2.095 (9)	C22—C23	1.360 (17)
Sb1—C19	2.115 (7)	C22—H22	0.9300
Sb1—O2	2.208 (5)	C23—C24	1.392 (14)
Sb2—O1	1.955 (5)	C23—H23	0.9300
Sb2—C31	2.096 (8)	C24—H24	0.9300
Sb2—C37	2.106 (8)	C25—C30	1.325 (13)
Sb2—C43	2.111 (7)	C25—C26	1.393 (14)
Sb2—O4	2.229 (5)	C26—C27	1.380 (12)
Cl1—C2	1.735 (9)	C26—H26	0.9300
Cl2—C8	1.730 (9)	C27—C28	1.340 (14)
N1—C6	1.317 (14)	C27—H27	0.9300
N1—C2	1.317 (11)	C28—C29	1.343 (14)
N2—C8	1.319 (10)	C28—H28	0.9300
N2—C12	1.336 (13)	C29—C30	1.400 (12)
O2—C1	1.261 (9)	C29—H29	0.9300
O3—C1	1.232 (9)	C30—H30	0.9300
O4—C7	1.251 (9)	C31—C32	1.358 (11)
O5—C7	1.244 (9)	C31—C36	1.360 (11)
C1—C3	1.498 (10)	C32—C33	1.372 (14)
C2—C3	1.376 (12)	C32—H32	0.9300
C3—C4	1.398 (11)	C33—C34	1.362 (16)
C4—C5	1.372 (14)	C33—H33	0.9300
C4—H4	0.9300	C34—C35	1.347 (14)
C5—C6	1.343 (15)	C34—H34	0.9300
C5—H5	0.9300	C35—C36	1.395 (13)
C6—H6	0.9300	C35—H35	0.9300
C7—C9	1.518 (10)	C36—H36	0.9300
C8—C9	1.374 (12)	C37—C42	1.356 (11)
C9—C10	1.388 (11)	C37—C38	1.369 (11)
C10—C11	1.364 (14)	C38—C39	1.386 (14)
C10—H10	0.9300	C38—H38	0.9300
C11—C12	1.361 (16)	C39—C40	1.357 (15)
C11—H11	0.9300	C39—H39	0.9300
C12—H12	0.9300	C40—C41	1.344 (13)
C13—C14	1.363 (10)	C40—H40	0.9300
C13—C18	1.373 (11)	C41—C42	1.391 (11)
C14—C15	1.369 (14)	C41—H41	0.9300
C14—H14	0.9300	C42—H42	0.9300
C15—C16	1.338 (16)	C43—C48	1.369 (11)
C15—H15	0.9300	C43—C44	1.371 (11)
C16—C17	1.361 (15)	C44—C45	1.382 (12)
C16—H16	0.9300	C44—H44	0.9300
C17—C18	1.372 (13)	C45—C46	1.355 (13)
C17—H17	0.9300	C45—H45	0.9300
C18—H18	0.9300	C46—C47	1.332 (13)
C19—C20	1.354 (12)	C46—H46	0.9300
C19—C24	1.395 (11)	C47—C48	1.377 (12)
C20—C21	1.376 (13)	C47—H47	0.9300
C20—H20	0.9300	C48—H48	0.9300
			
O1—Sb1—C13	90.9 (3)	C22—C21—C20	121.2 (12)
O1—Sb1—C25	91.9 (3)	C22—C21—H21	119.4
C13—Sb1—C25	126.1 (4)	C20—C21—H21	119.4
O1—Sb1—C19	97.0 (3)	C21—C22—C23	121.3 (11)
C13—Sb1—C19	114.7 (3)	C21—C22—H22	119.4
C25—Sb1—C19	118.3 (4)	C23—C22—H22	119.4
O1—Sb1—O2	179.2 (2)	C22—C23—C24	118.7 (12)
C13—Sb1—O2	89.2 (2)	C22—C23—H23	120.7
C25—Sb1—O2	88.7 (3)	C24—C23—H23	120.7
C19—Sb1—O2	82.2 (2)	C23—C24—C19	119.1 (11)
O1—Sb2—C31	95.1 (3)	C23—C24—H24	120.4
O1—Sb2—C37	93.9 (3)	C19—C24—H24	120.4
C31—Sb2—C37	114.9 (3)	C30—C25—C26	117.8 (9)
O1—Sb2—C43	92.7 (2)	C30—C25—Sb1	122.8 (8)
C31—Sb2—C43	128.1 (3)	C26—C25—Sb1	119.3 (8)
C37—Sb2—C43	115.6 (3)	C27—C26—C25	120.3 (11)
O1—Sb2—O4	174.6 (2)	C27—C26—H26	119.9
C31—Sb2—O4	88.4 (2)	C25—C26—H26	119.9
C37—Sb2—O4	80.8 (2)	C28—C27—C26	120.4 (11)
C43—Sb2—O4	88.3 (2)	C28—C27—H27	119.8
C6—N1—C2	115.8 (10)	C26—C27—H27	119.8
C8—N2—C12	115.8 (9)	C27—C28—C29	119.9 (10)
Sb1—O1—Sb2	165.1 (4)	C27—C28—H28	120.0
C1—O2—Sb1	116.1 (5)	C29—C28—H28	120.0
C7—O4—Sb2	124.1 (5)	C28—C29—C30	119.8 (11)
O3—C1—O2	125.6 (7)	C28—C29—H29	120.1
O3—C1—C3	119.3 (8)	C30—C29—H29	120.1
O2—C1—C3	114.9 (8)	C25—C30—C29	121.5 (11)
N1—C2—C3	126.8 (9)	C25—C30—H30	119.3
N1—C2—Cl1	113.1 (8)	C29—C30—H30	119.3
C3—C2—Cl1	120.0 (7)	C32—C31—C36	120.0 (8)
C2—C3—C4	113.9 (8)	C32—C31—Sb2	119.0 (7)
C2—C3—C1	127.2 (8)	C36—C31—Sb2	121.0 (6)
C4—C3—C1	118.7 (8)	C31—C32—C33	121.2 (10)
C5—C4—C3	120.5 (10)	C31—C32—H32	119.4
C5—C4—H4	119.7	C33—C32—H32	119.4
C3—C4—H4	119.7	C34—C33—C32	118.6 (10)
C6—C5—C4	118.1 (10)	C34—C33—H33	120.7
C6—C5—H5	120.9	C32—C33—H33	120.7
C4—C5—H5	120.9	C35—C34—C33	120.7 (11)
N1—C6—C5	124.6 (10)	C35—C34—H34	119.6
N1—C6—H6	117.7	C33—C34—H34	119.6
C5—C6—H6	117.7	C34—C35—C36	120.3 (11)
O5—C7—O4	126.7 (7)	C34—C35—H35	119.9
O5—C7—C9	116.2 (8)	C36—C35—H35	119.9
O4—C7—C9	117.1 (7)	C31—C36—C35	118.9 (9)
N2—C8—C9	125.5 (8)	C31—C36—H36	120.6
N2—C8—Cl2	113.8 (7)	C35—C36—H36	120.6
C9—C8—Cl2	120.5 (6)	C42—C37—C38	119.6 (8)
C8—C9—C10	117.2 (8)	C42—C37—Sb2	120.9 (6)
C8—C9—C7	124.5 (8)	C38—C37—Sb2	119.4 (7)
C10—C9—C7	118.3 (9)	C37—C38—C39	118.9 (10)
C11—C10—C9	118.0 (10)	C37—C38—H38	120.5
C11—C10—H10	121.0	C39—C38—H38	120.5
C9—C10—H10	121.0	C40—C39—C38	120.8 (10)
C12—C11—C10	120.2 (10)	C40—C39—H39	119.6
C12—C11—H11	119.9	C38—C39—H39	119.6
C10—C11—H11	119.9	C41—C40—C39	120.6 (10)
N2—C12—C11	123.2 (10)	C41—C40—H40	119.7
N2—C12—H12	118.4	C39—C40—H40	119.7
C11—C12—H12	118.4	C40—C41—C42	119.0 (10)
C14—C13—C18	118.9 (8)	C40—C41—H41	120.5
C14—C13—Sb1	120.4 (6)	C42—C41—H41	120.5
C18—C13—Sb1	120.7 (6)	C37—C42—C41	121.1 (8)
C13—C14—C15	121.0 (10)	C37—C42—H42	119.5
C13—C14—H14	119.5	C41—C42—H42	119.5
C15—C14—H14	119.5	C48—C43—C44	118.4 (8)
C16—C15—C14	119.8 (11)	C48—C43—Sb2	124.3 (6)
C16—C15—H15	120.1	C44—C43—Sb2	117.3 (6)
C14—C15—H15	120.1	C43—C44—C45	119.5 (9)
C15—C16—C17	120.3 (11)	C43—C44—H44	120.2
C15—C16—H16	119.9	C45—C44—H44	120.2
C17—C16—H16	119.9	C46—C45—C44	121.3 (10)
C16—C17—C18	120.5 (11)	C46—C45—H45	119.4
C16—C17—H17	119.7	C44—C45—H45	119.4
C18—C17—H17	119.7	C47—C46—C45	119.1 (9)
C17—C18—C13	119.4 (10)	C47—C46—H46	120.4
C17—C18—H18	120.3	C45—C46—H46	120.4
C13—C18—H18	120.3	C46—C47—C48	121.1 (10)
C20—C19—C24	121.2 (8)	C46—C47—H47	119.4
C20—C19—Sb1	122.5 (6)	C48—C47—H47	119.4
C24—C19—Sb1	116.3 (7)	C43—C48—C47	120.6 (9)
C19—C20—C21	118.5 (10)	C43—C48—H48	119.7
C19—C20—H20	120.8	C47—C48—H48	119.7
C21—C20—H20	120.8		

### Hydrogen-bond geometry (Å, °)

**Table:**

*D*—H···*A*	*D*—H	H···*A*	*D*···*A*	*D*—H···*A*
C17—H17···N2^i^	0.93	2.66	3.556 (14)	163
C39—H39···O5^ii^	0.93	2.63	3.544 (12)	167
C23—H23···O3^iii^	0.93	2.37	3.166 (12)	144
C23—H23···O3^iii^	0.93	2.37	3.166 (12)	144

Symmetry codes: (i) −*x*+1, −*y*, −*z*+1; (ii) *x*, *y*+1, *z*; (iii) *x*, *y*−1, *z*.
